# Case History of Centenarian—Cancer—Radium

**Published:** 1916-08

**Authors:** Evan O’Neill Kane

**Affiliations:** Kane, Pa.


### Case History of Centenarian—Cancer—Radium

EVAN O'NEILL KANE, M. D., Kane, Pa.

S. McK., referred by Dr. E. S. Briggs of Tidioute. He had suffered eighteen years from a cancer of the right orbit, the early history of which could not be definitely determined. At the time of examination, about ten months ago, the entire orbital region was involved, with complete destruction of the interior and extension of the disease well over onto the temple, nodular masses springing from the external border of the orbit and temporal ridge. The cavity had been eaten out so completely that an open space existed, extending far back. The side of the nose was commencing to be eroded, thickened and nodular. A remnant of the eyeball, thickened and degenerated, was drawn into the inner orbital angle. A foul and irritating discharge subjected the adjacent skin to considerable excoriation. Pain was so severe as to require recourse nightly to anodynes, and often during the day. His health was otherwise fairly good for so advanced an age. He was taken to Kane Summit Hospital Sept. 9, 1915, and put under radium treatment, being given 6700 milligram hours of radium element during the first week. A severe reaction took place, with marked constitutional disturbance. The site of treatment, its vicinity, and the entire cheek, temple, right side of the upper jaw and nose, became first dark red, then edematous, and finally covered by an extensive radium burn of first and second degrees. The hair of that side of the head and the whiskers fell out. The cancer tissue formed a deep gray slough.

Prominent among the constitutional symptoms during reaction were chills, anorexia, diarrhoea, albuminous scanty urine, and a broncho-pulmonary disturbance suggestive of broncho-pneumonia, with, for three days, some edema at the base of both lungs which made breathing difficult and was quite alarming. The reaction was protracted, lasting nearly three weeks and subsiding very slowly, both constitutionally and locally. The sloughs were peculiarly tardy in detaching, and the swelling did not subside completely for two months. Then healing began, first with a gradual filling of the cavity by granulation tissue, and thereafter at all points it reached a level with the surrounding skin epithelial proliferation advanced from the edges, at first quite rapidly, but this cicatrization ceased later and skin grafts were necessary to carry on the process. This took place satisfactorily everywhere, though slowly, save across the upper third of the orbital space, upon which a mucous covered, smooth, ulcerating surface persisted, owing to the impossibility of bringing the skin margin down to meet it. At times this would partially skin over, but the patient, who was very refractory, would tear off the dressings to scratch the sore with his nails, claiming it was "full of bugs." Otherwise sound healing everywhere was completed. No vestige of cancerous tissue remained anywhere at the date of dismissal, June 8, 1916, either in the original focus, or upon the temple and nose where extensions had previously existed. The outer half of the zygoma had been devitalized, either by the cancer or the action of the radium, and had to be detached and removed about four months after patient's treatment commenced. The newly formed skin where the radium burn had been was soft, pliant and of a healthy rose pink tint, but without a return of hair except around the margins. As soon as the constitutional symptoms subsided he ate well, slept well, and was free from pain (we were able to discontinue his anodynes a short time after his admission), and, save for an annoying formication, such as is frequent with elderly people, and which he insisted was due to the creeping of "bugs," was in excellent physical condition most of the time.

It is interesting to note that a fracture of the thigh in the upper third, with an overriding of about five centimeters, had been sustained at the age of 98, from which he recovered without impairment of function under the care of the hospital staff of the Warren Emergency Hospital; and also a Colles' fracture of the right arm at the age of 100, tended by the late Dr. Suggart of Tidioute, with good union and no subsequent loss of usefulness. His eyesight and hearing are but triflingly impaired and his mind is quite clear, he being able to talk intelligently, though readily fatigued by protracted conversation. McK. is a native of Ireland (Scotch Irish), coming to this country at the age of 39, and living an outdoor life as a farmer and teamster in the oil region of Crawford County, where he for a time conducted, in addition to his farming, a rough sort of hotel saloon.

He had three wives, all of whom he has outlived, and a numerous progeny. These latter are, nearly every one, scattered, dead and forgotten. No one cares anything for him, and he has no property to covet. What little he ever accumulated was lost through going security for a friend. He was never sick a day in his life, he affirms, save from his cancer and the above mentioned fractures. A religious man, always leading a moral and temperate life, generous and good natured, industrious and with great powers of physical endurance, possessing a fair education and sound judgment, he was well equipped for the battle of life: yet, though already embarked upon his second century, this venerable relic of past generations has achieved nothing worthy of mention; the fields of art, science and literature have, none of them, been enriched by him; no great fortune has been made; no political or social aspirations, if he ever had any, have been satisfied. He has amounted to nothing, and neglected and forgotten he is declining into innocuous desuetude, with only, like Methuselah, his length of years to place to his account,—

> "A life of nothing, nothing worth
> From that first nothing ere his birth
> To that last nothing under earth."

230 Clay Street.

---

**A Unique Murder Case With Application of New Law Governing Expert Testimony** is the title of an article by Dr. James W. Putnam, in the American Journal of Insanity, April 1916.

The case detailed is that of a Pole, about 43 years old, who killed a young man on the beach near Dunkirk, and then notified some men to send for the police. He then sat down and waited on the beach until the officers came, when he walked toward them, extending his hands and told them to put on the hand-cuffs, as he had killed a man. The only motive for the crime was that someone told him he must kill someone because his mother had been killed in the European War. This thought was ever present with him until the morning of the day of the murder when he said: "The time has come; I must kill someone."

Owing to the unusual circumstances of the crime the court appointed an expert under an act which was passed by the legislature of 1915, which provides that the court may appoint its own expert to examine a defendant accused of crime and report to the court, and either the people or the defendant may put the court's expert on the stand for examination, as either party may desire. It was under this act that Dr. Putnam was appointed by the court to examine the defendant, and he reported that the man knew the nature of his act; that he knew it was contrary to the laws of the state; but that he did not know the quality of the act, namely, that he was doing wrong, because he laid the responsibility on God, who told him to kill.

The jury rendered a verdict of acquittal on the ground of insanity and the defendant was committed to the State Institution for Insane Criminals at Matteawan, N. Y.

The act of 1915 appears to be the first legislation in this state providing for the designation of official experts in cases where the issue is insanity.—E. A. S.

